# Screening for sexually transmitted infections in high school students in the Kurdistan Region of Iraq: a school-based screening study

**DOI:** 10.3389/fpubh.2026.1854348

**Published:** 2026-07-27

**Authors:** Waleed Salih Rasheed, Waleed Khalid Ibrahim, Azad Jameel Hussein, Farhad Shahab Isamael, Nazik Abdulrahman, Deldar Morad Abdulah, Sagvan Ali

**Affiliations:** 1 Department of Public Health, College of Health and Medical Technology, Duhok Polytechnic University, Duhok, Iraq; 2 Public Health Department, College of Health and Medical Technology-Shekhan, Duhok Polytechnic University, Kurdistan Region, Iraq; 3 General Directorate of Health, Duhok, Iraq; 4 Community Health Nursing and Psychiatric Nursing Unit, College of Nursing, University of Duhok, Kurdistan Region, Iraq; 5 Duhok Directorate General of Health, Duhok, Iraq

**Keywords:** adolescents, hepatitis B virus, hepatitis C virus, high school students, human immunodeficiency virus, Kurdistan Region of Iraq, mass screening, sexually transmitted infections

## Abstract

**Introduction:**

Sexually transmitted infections (STIs) are a major, long-lasting problem and priority for public health all over the world, coming along with various health issues. This study identified the prevalence of STIs among high school students in the Kurdistan Region of Iraq (KRI) in 2025.

**Methods:**

The cross-sectional research was conducted on 591 high school students in the KRI as part of an STI screening program carried out by the Preventive Health Department.

**Results:**

The average age of the participants was 16.31 years (15–19 years), and there were slightly more women (57.2%) than men involved. 10th grade was the most populated grade, with 55.2% of students; almost all were Kurdish (98.7%) and Muslim (91.4%). In terms of risk behaviors, the majority did not have any surgery (86.5%) or dental work done (95.6%); none of them reported sharing needles or personal articles. Very few people had transfusions (0.9%), tattoos (1.0%), or piercings (0.5%). Use of illicit drugs was extremely rare (0.2%). Vaccination coverage against hepatitis B was very low (5.9%), with 81.6% of the population being unvaccinated. The results of the laboratory tests indicated that the prevalence of HBV was 0.7%, while HCV and HIV were not present. Only one case of syphilis was reported (0.2%).

**Conclusion:**

The results indicate that high school students in the Kurdistan Region of Iraq (KRI) have very low overall STI rates, with very few cases of HBV and syphilis and no HIV or HCV cases, coupled with minor involvement in high-risk behaviors. One of the concerns highlighted is the extremely low rate of HBV vaccination coverage.

## Introduction

Sexually transmitted infections (STIs) are a major global public health problem that affects millions of people around the world and has health, social, and economic implications (1, 2). The World Health Organization (WHO) predicts that over one million people get STIs every day, some of which are curable STIs, which account for approximately 333 million cases annually (2, 3). A quite frightening figure indicates that one out of every twenty teenagers is thought to get a curable STI each year, although this figure does not consider viral infections like the Human Immunodeficiency Virus (HIV), which are most common (3).

The global burden of STIs disproportionately affects adolescents and young adults. Defined as those aged 10 to 24 years, this demographic is responsible for up to 60% of all new infections worldwide, and even in the United States, where half of the STI cases are attributed to youth aged 15 to 24 annually (4). The high incidence of infections in this age group can be attributed to multiple factors, including biological, behavioral, and societal (5). Young women, for example, are more at risk of contracting infections like *Chlamydia trachomatis* owing to the presence of cervical ectopy, a condition that usually occurs among sexually active adolescents (6). The Adolescence is a critical time of quick biopsychosocial development, increased experimentation with sexuality, and stronger peer group influences, which often result in the adoption of unsafe sexual behaviors, such as unprotected sex and having numerous sexual partners (7).

Several primary factors explain the high rate of STIs among young people. One of the main obstacles to efficiently controlling STIs is the lack of adequate knowledge about their causes, symptoms, and prevention methods (4, 8). Studies repeatedly show that although the awareness of HIV/AIDS is fairly high, knowledge of other common STIs, their routes of transmission, and possible complications is very low (8, 9).

The lack of comprehensive sex education in schools and the prevailing social taboos regarding discussions of sexual health not only do not diminish this issue but have even worsened it by creating a knowledge gap (9). As a result, adolescents often defer or seek treatment late, sometimes because they do not recognize symptoms, sometimes due to potential stigmatization, and at other times due to fear and judgment regarding professional health care (10). The inconsistent use of condoms in adolescents, often attributed to inconvenience or a perceived reduction in sexual pleasure, further increases transmission risk (11).

Comprehensive epidemiological data, particularly about STIs, are limited and often poor in Iraq, especially regarding the Kurdistan Region of Iraq (KRI). Current data frequently depend on the syndromic approach or focus primarily on HIV/AIDS and do not represent a complete spectrum of infections (9). The KRI generally claims to have low HIV prevalence, often because cultural and religious norms prevent premarital sexual activity and encourage marital fidelity and the practice of male circumcision (12). However, other conditions may encourage conditions conducive to STIs. Ongoing political instability, displacement, poverty, unemployment, and fractured community organization continue, and these characteristics create a situation where STIs may have an easier opportunity to spread and be significantly underreported (9). More research specific to the region highlights a lack of information on the rates of genital warts in Iraqi Kurdistan; for instance, a study in Erbil City referencing the incidence quantity as low may be influenced by specific cultural inhibitions and male circumcision (13).

Moreover, knowledge gaps extend far beyond just the general populace; for example, secondary school teachers in Baghdad demonstrated very little knowledge of Human Papillomavirus (HPV) infection and HPV’s link to cervical cancer. Furthermore, many teachers did not know that STIs are a cause of cervical cancer (14). This highlights a broader educational deficit requiring urgent attention through targeted health campaigns to prevent a projected increase in cervical cancer cases (15). Given the global burden of STIs, the established vulnerability of adolescents, the identified risk factors, and the acute scarcity of comprehensive, current epidemiological data specifically about high school students in Duhok City within the Kurdistan Region of Iraq, this study addresses a critical information gap.

It is extremely important to identify the prevalence and the factors associated with STIs in this particular, usually underserved, demographic to allow the development of public health strategies that are based on evidence and that are culturally sensitive and efficient. The absence of such fundamental data makes it impossible to properly design or implement effective prevention, early detection, and treatment programs for the young population in the region. This study set out to establish the prevalence of STIs and infections in a group of high school students in Duhok City, which is located in the Kurdistan Region of Iraq.

## Materials and methods

### Study design

This cross-sectional research included blood samples obtained from 18 high school students residing in Duhok City, which is in the Duhok Governorate of the Kurdistan Region of Iraq. The study intended to include male and female students. The research was carried out by the Preventive Health Department of the Duhok General Directorate of Health and was part of a screening program targeting STIs among students. A group consisting of technicians and one researcher visited the selected schools for sample collection. Blood samples were drawn only after securing permission from the respective school authorities. The samples were then put into appropriate containers and sent to the Central Laboratory in Duhok City for processing and investigation.

The study included all eligible students of both genders, regardless of age or socio-demographic background. The sole reason for exclusion was not being available for the data collection.

### Settings and sampling technique

This research involved 591 secondary school students from Duhok City. High schools in this region cater to students from the seventh to the twelfth grade. A full list was acquired from the Duhok Governorate’s Directorate of Education to pinpoint the appropriate schools. Out of a total of 152 high schools—where secondary schools have been integrated into high schools for the past decade—we aimed to include approximately 10% of schools to achieve a representative sample size suitable for the large student population. All children present at the schools were eligible for inclusion in the study, and no exclusion criteria were applied to the participants. However, absent students were not contacted or included. In addition, because the program focuses on screening objectives, not all students in the schools were included in the study.

Students were selected from each chosen school using proportional allocation and systematic random sampling to ensure fair representation across schools. To enhance representativeness, the selected schools were increased to confirm coverage of areas with diverse socio-demographic characteristics, including variations in economic status. Data collection was carried out between February and March 2025.

The data for this study were collected by the Directorate of Preventive Health Affairs in Duhok province and are under the General Directorate of Health. The Directorate of Preventive Health Affairs is responsible for determining health policies and considering preventive health services and general administration of primary health care delivery systems. The Communicable Disease Department is responsible for the control and prevention of communicable infectious diseases, and the Sexually Transmitted Diseases (STDs) Unit operates within the CDD for screening, monitoring, and management of HIV, hepatitis B (HBV), hepatitis C (HCV), and syphilis, especially among high-risk groups.

Data collection was focused on assessing the possibility of infectious diseases, HIV, HCV, HBV, and syphilis among high school students. This study is a part of broader public health initiatives with the focus of disease prevention and control at the school level. This was also conducted as part of a screening program for students. Infectious disease screening is a principal function of the Communicable Diseases Department (CDD), as they routinely screen populations in high-risk and institutional settings such as prisons, among travelers, and at military bases.

The Preventive Health Department in Duhok Governorate has a continuous screening program in schools (all types of schools), prisons, and other vulnerable groups for communicable and infectious diseases, including STIs. The results reported in this study are the findings of the screening program conducted by health staff in this region. In this program, health staff visit either a sample of the population or, in some cases, the entire population to collect biological samples for different types of infections, including STIs.

### Measurements

Blood specimens from 591 students were collected by trained technicians and sent to the Central Laboratory in Duhok City for assessments. The screening was for the most prevalent STIs, including hepatitis B virus (HBV), hepatitis C virus (HCV), human immunodeficiency virus (HIV), and syphilis. In addition to the biological samples taken, two researchers collected general and medical information from the participants using a pre-designed questionnaire. The questionnaire included information regarding socio-demographic characteristics, the medical history of the individual before the study, previous laboratory confirmation of STIs, and potential reasons for the transmission of STIs (risk factors). Testing for the STIs was performed using the following laboratory procedures.

### Biological sampling and analysis

Blood specimens were obtained by trained technicians before being sent to the Central Laboratory in Duhok City for analysis. The serological tests allowed us to screen for the following STIs:

- *Hepatitis B (HBV)*: Detection of hepatitis B surface antigen (HBsAg) was performed using enzyme-linked immunosorbent assay (ELISA).
- *Hepatitis C (HCV)*: Presence of anti-HCV antibodies was determined using ELISA.
- *Syphilis*: Screening was conducted using the Venereal Disease Research Laboratory (VDRL) test to detect antibodies against *Treponema pallidum*.
- *HIV:* Initial screening was carried out using ELISA to identify HIV antibodies.

All positive results were confirmed following the standard diagnostic protocols recommended by the health authorities.

### Statistical analyses

The normality of the general information of the school children was confirmed using a Q-Q plot. The general and medical characteristics of the participants in the study were summarized by means (standard deviations) and frequencies (percentages) for the continuous and categorical variables, respectively. The age normality was determined with the help of a Q-Q plot and histogram visualization, which suggested that the age distribution was nearly normal. The STI prevalence, together with the related risk factors and previous STI cases, was computed and displayed as frequencies and percentages. All the statistical analyses were performed with the help of JMP® software, Version 18.0 (SAS Institute Inc., Cary, NC; 1989–2023).

### Ethical approval

The local health ethics committee of the Duhok Directorate General of Health in Duhok, Kurdistan Region of Iraq, granted ethical approval for the study. The study protocol received the number 08012025–1-50 and was registered on January 8, 2025. Relevant school administrations provided written permission for the data collection process. Participation was open, and people could decline to answer any general or medical questions. The blood samples were collected by skilled workers who observed strict health and safety rules. The study abided by the ethical guidelines laid out in the Declaration of Helsinki. The personnel who collected blood samples from the children had legal authorization to do so. The health staff of the Preventive Medicine Department work continuously with schools and other similar settings for screening purposes. In addition, school administrative staff inform parents that the health staff of the governorate are legally authorized to collect blood samples from children.

## Results

The students had an average age of 16.31 years, and the age range was from 15 to 19 years. Females had a slight majority (57.2%) over males and came from different grades. Almost all of the students were Kurdish (98.7%) and mainly Muslim (91.4%), while other students were Yazidi (8.3%) or Christian (0.3%; Table 1).

**Table 1:** General characteristics of the high school students in the Kurdistan Region of Iraq in 2024 (Duhok Governorate).

General characteristics (*n* = 591)		All patients	
		No.	%
Age (15–19 years)	Std Err Mean: 0.04	Mean: 16.31	SD: 0.86
Gender	Male	253	42.81
	Female	338	57.19
Education level	10th	326	55.16
	11th	141	23.86
	12th	124	20.98
Ethnicity	Kurd	583	98.65
	Arab	8	1.35
Religion	Muslim	540	91.37
	Yazidis	49	8.29
	Christian	2	0.34

A majority of students reported never undergoing surgical operations (86.5%) or dental procedures (95.6%). Importantly, none of the participants reported sharing needles or personal items such as razors or toothbrushes, eliminating some common transmission routes. Risky behaviors were low in school children. Vaccination coverage for hepatitis B was low; only 5.9% had received the vaccine, while the majority (81.6%) had not, and 12.5% were unsure (Table 2).

**Table 2:** STI-related information on high school students in the Kurdistan Region of Iraq in 2025.

STI-related information (*n* = 591)	Frequency distribution	
	No.	%
Tested or diagnosed with STIs		
No	583	98.65
Yes	8	1.35
Having any surgical operation		
No	511	86.46
Yes	80	13.54
Having dental procedures		
No	565	95.60
Yes	26	4.40
Sharing any needle, syringe, or other injection		
No	591	100
Having blood or blood product transfusions		
No	586	99.15
Yes	5	0.85
Having any tattoos		
Declined	2	0.34
No	583	98.65
Yes	6	1.02
Tattoos done in a professional setting		
Bladder Surgery	1	16.67
No	2	33.33
Yes	3	50.00
Having any body piercings		
Declined	3	0.51
No	585	98.98
Yes	3	0.51
Piercing done in a professional setting		
No	3	75.00
Yes	1	25.00
Having unprotected sex (without a condom)		
Declined	1	0.17
No	590	99.83
Administering illicit injection drugs		
Declined	3	0.51
No	587	99.32
Yes	1	0.17
Sharing any personal items (toothbrush or razor) with others		
No	591	100
Vaccinated against hepatitis B		
Do not know	74	12.52
No	482	81.56
Yes	35	5.92
Doses of received hepatitis B vaccine		
Do not know	9 (25.00)	
No	1 (2.78)	
One	2 (5.56)	
Two	7 (19.44)	
Three	14 (38.89)	
More than three	3 (8.33)	

The third table shows the laboratory results of STI prevalence. Hepatitis B virus (HBV) was detected in 0.7% of students, while hepatitis C virus (HCV) and human immunodeficiency virus (HIV) were absent in all cases. Syphilis was found in just one student (0.2%; Table 3).

**Table 3:** Prevalence of STIs in high school students in the Kurdistan Region of Iraq (Duhok Governorate).

STIs (*n* = 591)	Prevalence of STIs No. (%).	
	Negative	Positive
HBV	587 (99.32)	4 (0.68)
HCV	591 (100)	0 (0.0)
HIV	591 (100)	0 (0.0)
Syphilis (VDRL)	590 (99.83)	1 (0.17)

The percentage of students who had received dental procedures increased progressively with grade level, from 2.45% in the 10th grade to 5.67% in the 11th grade and 8.06% in the 12th grade (*p* = 0.0243), reflecting the age of the students as well (*p* = 0.0053). This implies that older students or those in higher grades had a greater likelihood of having received dental interventions, possibly indicating accumulated dental needs or greater access to care with age. In Table 4 no other association was seen.

**Table 4:** The associated factors to dental procedures in high school children.

Factors (*n* = 591)	Dental procedure		
	No	Yes	*p*
Age	16.29 (0.85)	16.77 (1.03)	0.0053
Gender			
Male	243 (96.05)	10 (3.95)	0.6468
Female	322 (95.27)	16 (4.73)	
Education grades			
10th	318 (97.55)	8 (2.45)	0.0243
11th	133 (94.33)	8 (5.67)	
12th	114 (91.94)	10 (8.06)	
Religion			
Christian	2 (100)	0 (0.00)	0.6676
Muslim	515 (95.37)	25 (4.63)	
Yazidi	48 (97.96)	1 (2.04)	

Chi-squared test was performed for statistical analyses.

## Discussion

The students were mainly 16-year-old Kurdish Muslims with a small female predominance. There was no history of risk behaviors such as needle sharing and low surgical/dental history. There was a low coverage of hepatitis B vaccination. STIs were very low in prevalence, with some cases of HBV and only one syphilis case. Likelihood of dental procedures increased alongside grade level. This study addresses a critical gap in regional health data, as comprehensive information on STI prevalence and associated factors among adolescents in this specific demographic is notably scarce. Adolescents and young adults (typically 10–24 years) are globally recognized as a particularly vulnerable group for STIs, accounting for a significant proportion of new infections worldwide due to a complex interplay of biological, behavioral, and societal factors (4, 16). Understanding the local context, including sociodemographic characteristics, risk behaviors, and actual prevalence, is therefore crucial for developing targeted and effective public health interventions.

The age of the children in this study presents a period of major biopsychosocial growth and experimentation with sexuality. The majority of the participants were females, which is in agreement with the gender distribution in other school-based studies in different areas (7, 17, 18). The composition of the student body was 98.7% Kurdish and 91.4% Muslim, which mirrors the cultural and religious profile of the KRI. This ethnoreligious context is very significant, as deep-rooted cultural and religious values in the area usually discourage sex outside marriage and support marital faithfulness, thus affecting the sexual behaviors and the STI incidence reporting (19, 20).

The study, which involved a careful investigation into risk-taking behaviors and risk exposures, discovered an astonishingly low prevalence of some of the most common modes of STD transmission. No single student admitted to using needles or sharing personal items like razors or toothbrushes. These items are known to be carriers of blood-borne infections. Not reporting sharing needles and other direct contact/ blood-borne risk factors showing very low rates may be seen as a positive sign and a factor leading to the low prevalence of certain STIs, which was observed. Nevertheless, it is necessary to point out that self-reported data on sensitive matters, especially in conservative societies such as the KRI, could be affected by the social desirability bias and underreporting (19, 21, 22). It is possible that students are not willing to admit to doing something that is considered socially unacceptable, which in turn might cause the actual risk to be underestimated.

In spite of the reported low-risk behaviors, there was a remarkable finding of low hepatitis B vaccination coverage. The students’ vaccination status indicated that only 5.9% of them were vaccinated against HBV, while a staggering 81.6% were unvaccinated, and 12.5% were in doubt about their vaccination status. The lack of vaccination among students is very serious, as it means that a considerable number of them could easily contract the virus through sexual or blood-to-blood transmission. Even the vaccinated students not only had doubts about their vaccination status but also about the exact number of doses they had received. This points to either a gap in health literacy or poor record-keeping. It is necessary to conduct awareness campaigns on the necessity of HBV vaccination, particularly as a preventive measure against sexually transmitted diseases, because of the low reported risk behaviors and the vaccination of a small percentage of the population.

The laboratory testing for STI prevalence showed that the high school students in Duhok City had a very low burden of STIs. All tested cases showed no presence of Human Immunodeficiency Virus (HIV) or HCV. This HIV finding parallels previous surveys that reported a low global incidence of HIV in Iraq and KRI, which is mainly due to strong cultural and religious norms (23). However, the low reported prevalence may also be attributable to under-reporting or limited testing, especially in light of the prevailing issues of geopolitical instability and community disintegration that might allow STIs to spread unnoticed (9).

Among the students, Hepatitis B Virus was detected in only a small fraction (0.7%), and syphilis was reported in one person only (0.2%). These figures are very low, especially when compared to the prevalence rates of other developing countries or even certain groups of people in the KRI who are considered vulnerable. For example, a study on prisoners in Duhok City, although predominantly male and older, revealed a syphilis prevalence of 0.59% (22). The ranges found in the studies among blood donors in Iraq were from 0.21 to 0.36% for the prevalence of syphilis (24) The very low prevalence rate of HBV in the present study, despite the very low vaccination rate, indicates that students might be biologically susceptible but their actual exposure to the virus might be limited by other factors, possibly including conservative sexual practices that are not fully captured in the questionnaire. The very low percentage of detection of syphilis in just one student signifies that, even in low-prevalence settings, these infections can happen. Adolescents typically do not experience the worst outcomes of syphilis, such as congenital syphilis or tertiary syphilis (3).

Comparing these results with studies from other regions highlights the unique context of Duhok. For example, self-reported STI prevalence among sexually active university students in Northeast Ethiopia was 21.5% (25). In Serbia, approximately 18.0% of high school students reported STI symptoms (21). In Nigeria, some studies reported candidiasis prevalence around 10.9% and gonorrhea at 0.6% among adolescents (7). Globally, significant proportions of adolescents and young people are affected by chlamydia and gonorrhea, with prevalence rates in some studies reaching over 10% (3). The current study’s findings of zero prevalence for HIV/HCV and extremely low rates for HBV/syphilis are markedly lower than many international reports, suggesting that the specific sociocultural context and potentially lower rates of risky sexual behaviors among high school students in Duhok play a protective role. Local studies in Erbil City on genital warts also indicated a low incidence, possibly due to cultural inhibitions and male circumcision, further supporting a generally low STI prevalence in the KRI that might be influenced by social factors (20).

The low prevalence of STIs in the study sample, while encouraging, should nonetheless be interpreted with caution, and at the same time, the need for sexual health education and comprehensive prevention strategies should not be overlooked. Data worldwide confirm that a lot of adolescents and young adults have inadequate and inaccurate knowledge about STIs, their symptoms, and transmission pathways (8, 17, 26). For example, a survey conducted at Baghdad University revealed that 78% of the respondents had a poor understanding of STIs, with the students from non-medical faculties exhibiting the most severe deficits in understanding. The secondary school teachers in Baghdad also had a very limited knowledge of HPV and its relationship with cervical cancer, and they even considered STIs as not being a causative factor (14). Misunderstandings regarding STI transmission, like getting them from toilets or through sharing clothes, are also rampant among high school students in different countries (8, 17, 27).

Considering the low HBV vaccination rates and the overall knowledge gap in similar Iraqi populations, it is very likely that the students of Duhok City have low knowledge of STIs, limited to HIV/AIDS only. The educational gap is more serious because the absence of comprehensive sex education in many secondary school curricula, along with the prevailing societal taboos around sexual health, prevents effective preventive measures from being established (7, 26). Gaps in knowledge lead to treatment being delayed, and this is particularly the case when treatment-seeking is not only based on one’s recognition of symptoms but also on fear of being judged and concerns about confidentiality and stigma (3, 7, 21). A person may not even attempt to get treated even after being diagnosed with STIs due to poor perception of what to do, and some even think relying on divine intervention or homebrew remedies is the way to go (7). Besides that, the absence of youth-friendly clinics makes it even more difficult for young people to get the right care (17).

The results of the study, particularly the low prevalence against the backdrop of sexual activity that might not have been measured and the documented knowledge gaps, imply several things. First of all, even though direct high-risk sexual behaviors may seem low or might be underreported, the low HBV vaccination rate still represents a risk that can be prevented and should therefore be dealt with through targeted public health campaigns. Secondly, the lack of reported HIV and HCV cases is encouraging, but it still calls for monitoring and possibly even more extensive screening if the patterns of sexual activity were to change. Thirdly, the research points out the critical requirement for a culturally sensitive and complete sexual health education in schools in Duhok City and the KRI. This education should not only inform about HIV but also cover a wider spectrum of STIs, their symptoms, methods of transmission (including non-sexual for HBV), prevention strategies (including vaccination), and the necessity of timely, evidence-based medical care (17, 27, 28). Ideally, supported by health professionals, such programs can alter misconceptions, create acceptance regarding sexual health, and take away the stigma related to STIs (1, 26).

### Strengths and limitations

The main strength of this study is the successful random sampling process and sufficient sample size. However, the generalizability of the findings may be limited to the wider region because of the participation of schoolchildren from one city in the Kurdistan Region. Therefore, a multi-center study would provide a more complete picture of STI prevalence within this population.

### Clinical or practical implications

The KRI research should adopt methods in the future that will be able to collect sensitive behavioral data more effectively, respecting cultural norms, possibly through qualitative methods or anonymous surveys with very advanced confidentiality measures. This would give a better understanding of the hidden sexual behaviors and risk factors that are hard to assess directly in studies among adolescents (19).

## Conclusion

In summary, the results suggest a relatively low current burden of sexually transmitted and blood-borne infections among high school students in the Kurdistan Region of Iraq. From a public health perspective, this situation should not lead to complacency. Gaps in preventive coverage, particularly low uptake of hepatitis B vaccination and incomplete awareness of vaccination status, could increase vulnerability in the future. Strengthening school-based health education, promoting preventive behaviors, and expanding immunization programs are essential to keep the risk of transmission low and prevent future emergence of infection in this population.

## Data Availability

The original contributions presented in the study are included in the article/supplementary material, further inquiries can be directed to the corresponding author.
